# LRP receptors in chondrocytes are modulated by simulated microgravity and cyclic hydrostatic pressure

**DOI:** 10.1371/journal.pone.0223245

**Published:** 2019-10-04

**Authors:** Rachel C. Nordberg, Liliana F. Mellor, Andrew R. Krause, Henry J. Donahue, Elizabeth G. Loboa

**Affiliations:** 1 College of Engineering, University of Missouri, Columbia, Missouri, United States of America; 2 Spanish National Cancer Research Centre, Madrid, Spain; 3 Sport Health and Physical Education, Vancouver Island University, Nanaimo, British Columbia, Canada; 4 Division of Musculoskeletal Sciences, Department of Orthopaedics and Rehabilitation, Penn State College of Medicine, Hershey, Pennsylvania, United States of America; 5 Department of Biomedical Engineering, Virginia Commonwealth University, Richmond, Virginia, United States of America; University of Umeå, SWEDEN

## Abstract

Mechanical loading is essential for the maintenance of musculoskeletal homeostasis. Cartilage has been demonstrated to be highly mechanoresponsive, but the mechanisms by which chondrocytes respond to mechanical stimuli are not clearly understood. The goal of the study was to determine how LRP4, LRP5, and LRP6 within canonical Wnt-signaling are regulated in simulated microgravity and cyclic hydrostatic pressure, and to investigate the potential role of LRP 4/5/6 in cartilage degeneration. Rat chondrosacroma cell (RCS) pellets were stimulated using either cyclic hydrostatic pressure (1Hz, 7.5 MPa, 4hr/day) or simulated microgravity in a rotating wall vessel (RWV) bioreactor (11RPM, 24hr/day). LRP4/5/6 mRNA expression was assessed by RT-qPCR and LRP5 protein expression was determined by fluorescent immunostaining. To further evaluate our *in vitro* findings *in vivo*, mice were subjected to hindlimb suspension for 14 days and the femoral heads stained for LRP5 expression. We found that, *in vitro*, LRP4/5/6 mRNA expression is modulated in a time-dependent manner by mechanical stimulation. Additionally, LRP5 protein expression is upregulated in response to both simulated microgravity and cyclic hydrostatic pressure. LRP5 is also upregulated *in vivo* in the articular cartilage of hindlimb suspended mice. This is the first study to examine how LRP4/5/6, critical receptors within musculoskeletal biology, respond to mechanical stimulation. Further elucidation of this mechanism could provide significant clinical benefit for the identification of pharmaceutical targets for the maintenance of cartilage health.

## Introduction

Mechanical loading is essential for the maintenance of musculoskeletal homeostasis. It is well known that mechanical loading stimulates bone formation but the absence of loading, such as in patients on prolonged bed-rest or astronauts on long-term space missions, leads to loss of bone mass [1–3] and skeletal muscle [4,5]. Cartilage has also been demonstrated to be highly mechanoresponsive. Similar to bone, insufficient loading can lead to cartilage degeneration. Patients on bed-rest experience loss of cartilage thickness after only 14 days [6]; and, muscle weakness has been associated with the progression of osteoarthritis [7,8]. However, excessive repetitive loading has been associated with chondrocyte death and cartilage degeneration [9,10]. Obesity is recognized as a major risk factor for osteoarthritis, in part due to increased axial loading patterns on the hip and knee joints [11]. Other studies suggest that moderate loading patterns from normal daily activities such as walking promote cartilage health [12–14]. The responses and the mechanisms by which chondrocytes respond to mechanical stimuli remains an active area of investigation.

The effects of mechanical loading and unloading on cartilage biology have been studied via hydrostatic pressure [15–18] and simulated microgravity [19–22], respectively. Cyclic hydrostatic pressure within physiologic magnitudes (<10 MPa) has been demonstrated to promote cartilage matrix deposition [15] and chondrogenesis in human bone-marrow derived mesenchymal stem cells [23–26] and human adipose-derived stem cells [22,27–29]. Cyclic hydrostatic pressure mimics physiologic loading patterns, which are necessary to prevent cartilage degeneration from disuse [12]. Simulated microgravity has been used to study chondrocytes and chondrogenesis in unloaded conditions [19–22]. Simulated microgravity can be produced by rotating wall vessel (RWV) bioreactors developed by NASA, which rotate at a constant speed to maintain pellets in free-fall resulting in a randomized gravitational vector [30]. The forces generated by this vessel produce vector-averaged forces comparable with that of near-earth free fall orbit [30,31]. Currently, however, it is unclear whether simulated microgravity promotes [19] or inhibits [20,22] cartilage matrix synthesis. *In vivo*, the hindlimb suspension rodent model was developed to mimic the microgravity environment experienced during space flight [32]. This model allows for preliminary studies to be carried out on Earth without taxing the limited resources available for spaceflight experimentation. While hindlimb suspension studies have primarily focused on bone-related research, recently hindlimb suspension was demonstrated to protect against articular cartilage degeneration in a rat osteoarthritis model [33]. Current literature clearly demonstrates that mechanical stimulation modulates cartilage biology, however the mechanisms by which cyclic hydrostatic pressure and simulated microgravity modulate chondrocytes need to be further elucidated.

Cartilage and bone are developmentally linked through processes such as endochondral ossification and share common signaling pathways including canonical Wnt-signaling. In active Wnt-signaling, Wnts bind to the frizzled and low-density lipoprotein receptor-related protein (LRP) co-receptors. This activates a signaling cascade, preventing β-catenin from degrading within the cytoplasm, thus allowing β-catenin to translocate and accumulate within the nucleus [34]. Nuclear β-catenin initiates the transcription of many genes responsible for bone and cartilage homeostasis [34–36]. LRP receptors are well known to be major mediators of musculoskeletal homeostasis. LRP5 and LRP6, specifically, are critical for transduction of canonical Wnt-signaling [35]. In bone, LRP5/6 receptors have been shown to be essential for maintaining balance between bone formation and resorption [3]. LRP receptors are also important for signaling in cartilage and, as in bone, have been indicated to transduce Wnt signaling and induce nuclear β-catenin localization. LRP5 has been found to be upregulated in osteoarthritis [37]. However, LRP6 loss-of-function mutation has been associated with an increased progression of osteoarthritis [38]. LRP4 has been implicated in regulation of Wnt-signaling, and has been reported to induce extracellular matrix production in cartilage [39]. However, the exact mechanism by which LRP4 acts within cartilage biology remains elusive, as it appears to have a role distinct from LRP5/6. In the context of mechanobiology, it has been demonstrated that tensile strain and Wnt3a act synergistically to activate Wnt signaling and trigger genes associated with cartilage catabolism [40]. Chondrocyte response to mechanical loading has been shown to be dependent on Wnt-signaling [41]. Furthermore, Wnt-signaling has been demonstrated to be activated following mechanical injury of cartilage [42]. However, it is currently unclear how LRP receptors respond to mechanical stimulation.

The goal of this study was to determine if, and to what extent, LRP receptors are regulated in simulated microgravity and cyclic hydrostatic pressure in order to elucidate the mechanisms by which mechanical stimulation regulates cartilage homeostasis. Since canonical Wnt-signaling and LRPs are involved in the maintenance of bone homeostasis and LRPs have been associated with cartilage pathologies, we hypothesized that LRP expression is responsive to these mechanical stimuli.

## Materials and methods

### Rat chondrosarcoma cell culture

Rat chondrosarcoma cells (RCS), which are a cell line derived from Swarm rat chondrosarcoma [43] were sent to us from collaborators at Boise State University who specialize in RCS culture (see acknowledgements). This cell type has been used extensively to study cartilage and cartilage matrix biology [44–46] and has been used to study chondrocyte response to mechanical stimulation [47]. RCS were cultured in Dulbecco's Modified Eagle Medium (DMEM) supplemented with 10% v/v fetal bovine serum, 100 units/ml penicillin, and 100 μg/ml streptomycin (RCS media). When pellet culture was utilized, RCS were resuspended at a density of 250,000 cells per pellet in 15 ml conical tubes. The RCS were centrifuged at 300 g for 5 min to form a pellet, and incubated for 2 days with loose tube caps in order for the cell pellets to coalesce.

### Stimulation in simulated microgravity and cyclic hydrostatic pressure

Pellets were subjected to either simulated microgravity or cyclic hydrostatic pressure and compared to control pellets, which were continuously incubated at 37°C, 5% CO_2_ without mechanical stimulation. The intent of using cyclic hydrostatic pressure in this experiment was to mimic physiological loading on the chondrocytes. Simulated microgravity was achieved by culturing cell pellets in a RWV bioreactor. The RWV bioreactor was set to a speed of 11RPM and kept in a standard 37°C, 5% CO_2_ incubator for the full 14 days. Any air bubbles that accumulated within the vessel were removed daily.

For cyclic hydrostatic pressure stimulation, the pellets were placed in sterile heat-sealed bags with 10 ml of RCS media and loaded in a custom cyclic hydrostatic pressure system designed by our lab, as described previously [28,48]. Briefly, the heat-sealed bags were placed in a stainless steel pressure vessel (Parr Instruments, Moline, Illinois) filled with mineral oil. The pressure vessel was connected via high-pressure stainless steel tubing to a hydraulic cylinder mounted to an MTS 858 Mini Bionix II load frame. Loading operations were controlled using a MTS TestStar control program (MTS System Corp, Eden Prairie, MN). Loading was applied at a magnitude of 7.5 MPa and frequency of 1Hz, 4 hours per day for up to 14 days. After daily loading, the heat-sealed bags were removed from the apparatus and maintained in a standard incubator at 37°C. Pellets were transferred to new bags with fresh media every 3–4 days.

### Mouse hindlimb suspension procedures

In order to further examine the effect of unloading *in vivo*, hindlimb suspension was used to unload the synovial joints of male C57BL/6J mice (Jackson Laboratories, Bar Harbor, ME) for 14 days. The protocol was performed in collaboration with the Penn State College of Medicine. All procedures were approved by the Penn State Institutional Animal Care and Use Committee (Protocol #2013–033) and were performed according to institutional guidelines. All mice in the study were randomly assigned to experimental groups. All the samples were analyzed blind; that is none of the investigators knew which experimental group a particular sample came from. All mice used were approximately 18 weeks old ± 5 days and thus skeletally mature on day 0 (start of experiment). WT mice weighed 30.7±0.2 grams and Sost KO weighed 31.0±0.3 grams at the start of experiment. There were four experimental groups and 8–9 mice per group for a total of 32–36 mice. The numbers were based on previous experiments demonstrating that 8 mice per group were sufficient to detect significant differences in micro architectural features at p<0.05. All mice in the study were randomly assigned to experimental groups. The order that the animals were treated and assessed was random and blinded. Sclerostin deficient male C57BL/6J mice (Sost-/-) were generated using a LacZ replacement of the sclerostin gene as described previously [49]. Mice were housed in standard enclosures modified for hindlimb suspension (2 mice/cage), with the room temperature at 25°C and on a 12-hour light/dark cycle. Corn cobb bedding with a wire floor above was used. The heath of the mice was assessed by Penn State Hershey veterinary staff prior to the study. During the study the health of the mice was assessed twice daily by Donahue Lab staff and daily by the veterinary staff at Penn State Hershey. Any health issues were immediately reported to the PI Dr. Donahue. Standard rodent 2018 Tekland Global 18% protein rodent diet (Harlan Laboratories Inc., Indianapolis, IN, USA) was provided ad libitum throughout the study. Mice were acclimated to the room used for hindlimb suspension for 1 week prior to study commencement. Hindlimb suspension mice were anesthetized and tape strips were secured to the tails. The tape allowed the animal to be attached to tethers that ran along the top of the cage. The tethers were adjusted to support the mouse at 30° of elevation. Ground control mice were kept in identical housing units without the tethers. After 14 days, mice were anesthetized with isoflurane (3% + oxygen) during final tissue collection, and then euthanized with isoflurane overdose (greater than 5% isoflurane concertation until 1 minute after breathing stops); confirmation of euthanasia was done via bilateral thoracotomy.

### Immunofluorescence

After 14 days of culture, RCS pellets were fixed in 10% buffered formalin for 1 hour (n = 4 simulated microgravity, n = 3 control, n = 3 cyclic hydrostatic pressure). After fixation, gross pellet morphology was imaged on an EZ4D histology microscope (Leica, Wetzlar, Germany). In addition, mouse femoral heads from control and hindlimb suspension mice (n = 3 or greater for all groups) were fixed in formalin for 36 hours and decalcified in a solution of 2.5% EDTA in 0.2 M phosphate-buffer, pH 6.0, as described previously [50]. Fixed pellets and femoral heads were paraffin embedded and sectioned at the North Carolina State University College of Veterinary Medicine histology facilities. Sections were taken at a thickness of 10 μm from pellets and mouse femoral heads for immunofluorescent staining. Both pellet and femoral head sections were deparaffinized in SafeClear II (Thermo Fisher, Waltham, MA), hydrated in ethanol series, and treated with Antigen Retrieval Reagent-Universal (R&D Systems, Minneapolis, MN) following manufacturer’s protocol. The samples were then blocked with a 0.2% v/v Triton X-100/5.0% v/v BSA stock solution for 40 minutes.

Primary antibody dilutions were prepared in a PBS solution containing 0.2% v/v Triton X-100, and 0.5% v/v BSA. Pellets were incubated in goat polyclonal antibody to LRP5 (1:500 dilution, Abcam, Cambridge, United Kingdom) and mouse monoclonal antibody to active β-catenin (1:300 dilution, Millipore, Billerica, MA). The mouse femoral head sections were incubated in a goat polyclonal antibody to LRP5 solution (1:500 dilution). Sections were incubated overnight at 4°C in the primary antibody solution and then rinsed three times in PBS. Secondary antibody solutions were prepared using chicken anti-mouse Alexa Fluor 488 (1:1000 dilution, Molecular Probes, Eugene, Oregon), donkey anti-goat 594 (1:1000 dilution, Molecular Probes), and DAPI (1:1000, Molecular Probes, Eugene, Oregon). The samples were incubated in secondary antibody solutions for one hour at room temperature followed by three more PBS washes. Prolong Gold Mounting Media (Molecular Probes, Eugene, Oregon) was used to mount coverslips on the slides. The slides were dried in the dark for 24 hours and imaged on a Leica DM5500B Fluorescent Microscope using the compatible LAS-AF software.

### Application of Wnt-regulating treatments

In order to determine if sclerostin levels alter LRP expression in RCS cells, RCS cells were cultured in RCS medium supplemented with recombinant sclerostin protein (R&D Systems, Minneapolis, MN). RCS were seeded at a density of 375,000 cells per well in standard six well plates and were incubated for 24 hours in RCS media. Media formulations were then switched to contain 25ng/ml, 100ng/ml, or 250ng/ml sclerostin protein and cultured for an additional 48 hours (n = 3 per condition).

### Total RNA extraction and RT-PCR

PCR was used to evaluate LRP expression after 3, 7, 10, and 14 days of exposure to simulated microgravity, cyclic hydrostatic pressure, or six-well plate control and after sclerostin stimulation experiments described above (n = 3 per condition). RNA extraction was carried out using a Trizol (Invitrogen, Carlsbad, CA) extraction method following the manufacturer’s protocol. RNA concentration and quality were assessed using a NanoDrop spectrophotometer (Thermo Scientific, Wilmington, DE). RNA was reverse-transcribed using Marligen’s First-strand cDNA Synthesis System (Origene, Rockville, MD). RT-PCR was performed with an ABI Prism 7000 system (Applied Biosystems, Carlsbad, CA) using SYBR Green (Life Technologies, Grand Island, NY) for fluorescent detection. Primers were designed using the Integrated DNA Technologies (Coralville, IA) website. RT-PCR data were analyzed using the 2^-ΔΔCT^ method [51] and 18s was used as the housekeeping control. Primer sequences used were: **18s** 5’AAGACGAACCAGAGCGAAAG3’, 3’TCTATGGCAGCATCAAGGCT5’; **LRP4** 5’GCAGCAAGAGGAAGGTACTAAT3’, 3’TCATAGGTTTCACGACTGGC5’; **LRP5** 5’CCATACAGGCCCTACATCATTC3’, 3’GATGGACCTGAACTTAAGCCTG5’; **LRP6** 5’GGGAGAAGTGCCAAAGATAGAA3’, 3’CTAATACTCCTCGCCTTCGAA5’.

### Statistical analyses

All gene expression data sets were analyzed in Statistical Package for the Social Science (IBM SPSS Statistics, North Castle, NY). Data were analyzed using Tukey test with p-values less than 0.05 considered statistically significant. All data are presented as averages with error bars representing standard error of the mean. All experiments were carried out with n = 3 or greater. A connecting letters report was used to denote statistical significance, and in each graph, levels not connected by the same letter are significantly different (p<0.05).

## Results

### Pellet growth was modulated by mechanical stimulation

**Fig 1:**
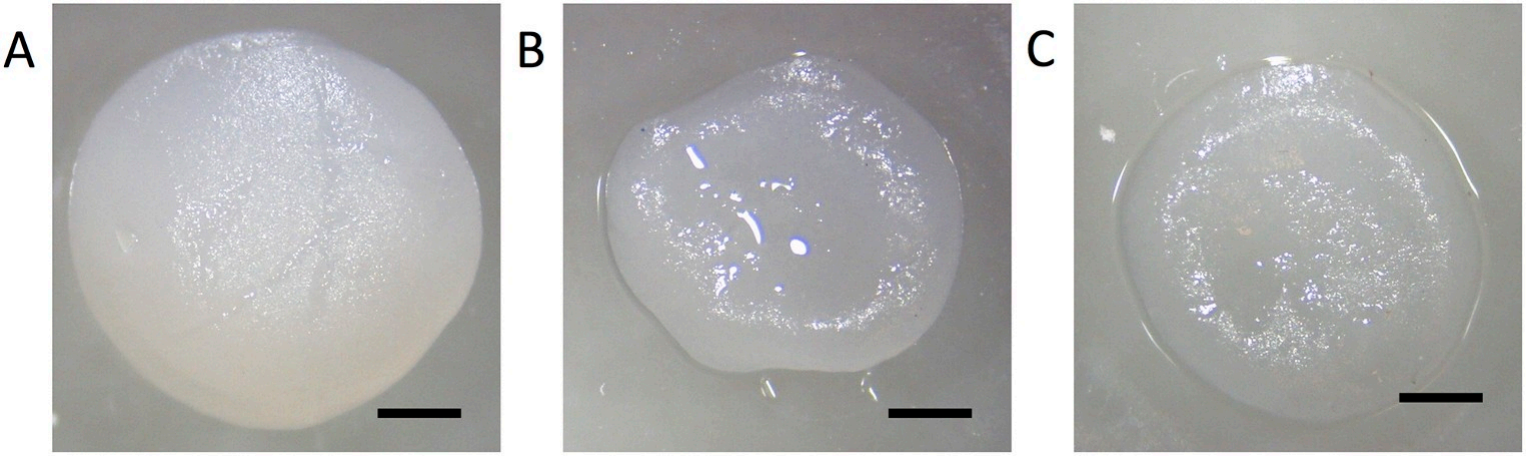
Gross morphology of pellets. Rat chondrosarcoma (RCS) pellets at day 14 in (**A**) simulated microgravity (11RPM, 24hrs/day) (**B**) control (six well plate, 37C, 5% CO2) and (**C**) cyclic hydrostatic pressure conditions (7.5 MPa, 1 Hz, 4 hrs/day) (Scale bars = 1mm).

Gross examination of the pellets showed that pellets in simulated microgravity grew larger than those in control and cyclic hydrostatic pressure conditions after 14 days in culture (Fig 1). In addition, pellets stimulated via cyclic hydrostatic pressure and simulated microgravity developed into circular disks, but the control pellets were more irregular in shape.

### LRP expression patterns are modulated by simulated microgravity and cyclic hydrostatic pressure

**Fig 2:**
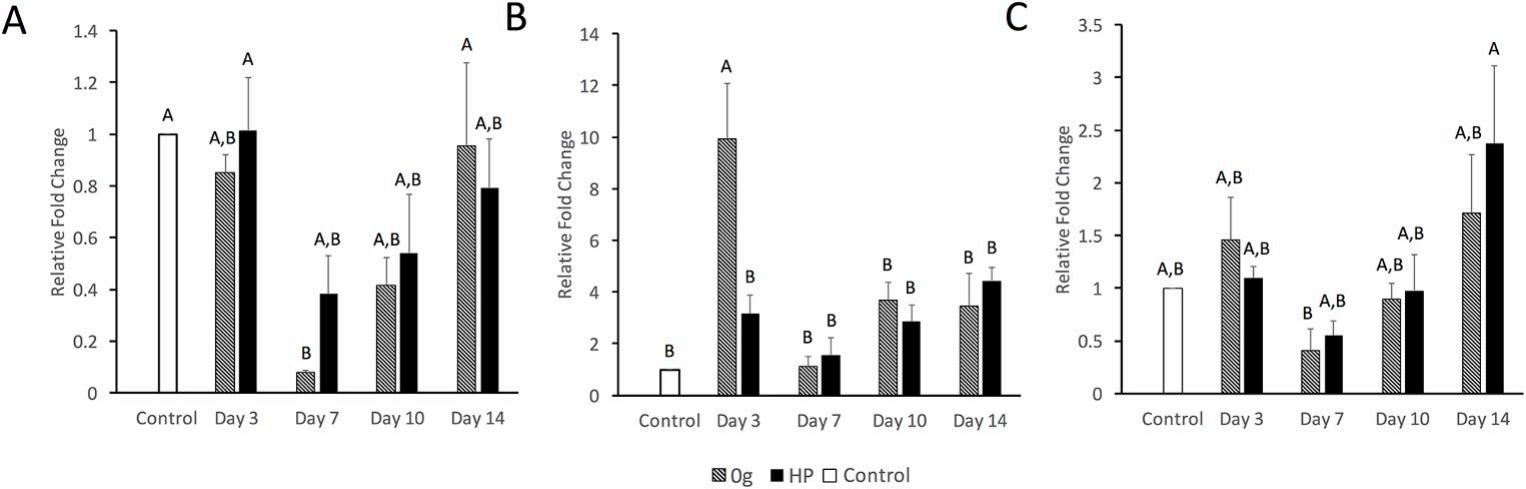
Gene expression of LRP4/5/6 in response to mechanical stimuli. Expression changes of (**A**) LRP4, (**B**) LRP5, and (**C**) LRP6 in response to simulated microgravity or cyclic hydrostatic pressure relative to unstimulated controls at days 3, 7, 10, and 14. All data was normalized to day 0 control data represented by the dashed line. Levels not connected by the same letter are significantly different (p<0.05).

Expressions of LRP4, LRP5, and LRP6 in response to simulated microgravity or cyclic hydrostatic pressure were evaluated at days 3, 7, 10, and 14 via quantitative RT-PCR (Fig 2) relative to control pellets. LRP4 mRNA expression was significantly reduced at the day 7 in simulated microgravity. LRP6 mRNA exhibited a 2.4 fold expression elevation in cyclic hydrostatic pressure at day 14 relative to controls. LRP5 mRNA expression was significantly upregulated in simulated microgravity at day 3 (9.9 fold). In all cases, LRP4/5/6 expression was lower at 7 than at day 3.

**Fig 3:**
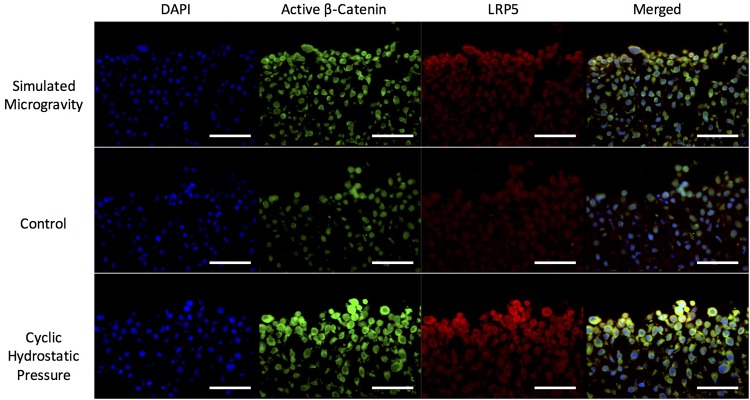
Immunofluorescence of LRP5 and β-catenin in response to mechanical stimuli. LRP5 and active β-catenin after 14 days of culture in simulated microgravity, unstimulated control (6-well plate, no loading) or cyclic hydrostatic pressure. All scale bars = 50 μm.

Since LRP5 was the most highly upregulated LRP within our RT-PCR data and has been demonstrated to be the LRP receptor most responsible for Wnt-mediated osteoarthritic cartilage destruction [52], we next evaluated LRP5 protein-level changes within the Wnt-signaling pathway in response to cyclic hydrostatic pressure and simulated microgravity. 14-day RCS pellets were stained for LRP5 and β-catenin with an antibody that recognizes the active form of β-catenin dephosphorylated on Ser37 or Thr41 (Fig 3). Congruent with our mRNA results, LRP5 protein expression in RCS cells was elevated in response to both simulated microgravity and cyclic hydrostatic pressure when compared to the unstimulated controls. LRP5 expression was greater in response to cyclic hydrostatic pressure than simulated microgravity. Active β-catenin followed the same trend as LRP5 protein expression with highest expression in RCS cells exposed to cyclic hydrostatic pressure and lowest expression in the unstimulated controls. All RCS cells stained for LRP5 and active β-catenin to some extent, although the intensity was greater in the cells that had been subjected to mechanical stimulation. It should also be noted that cells that were stimulated via mechanical stimulation were larger in size than the unstimulated controls, which enhanced the overall appearance of LRP5 and active β-catenin expression.

**Fig 4:**
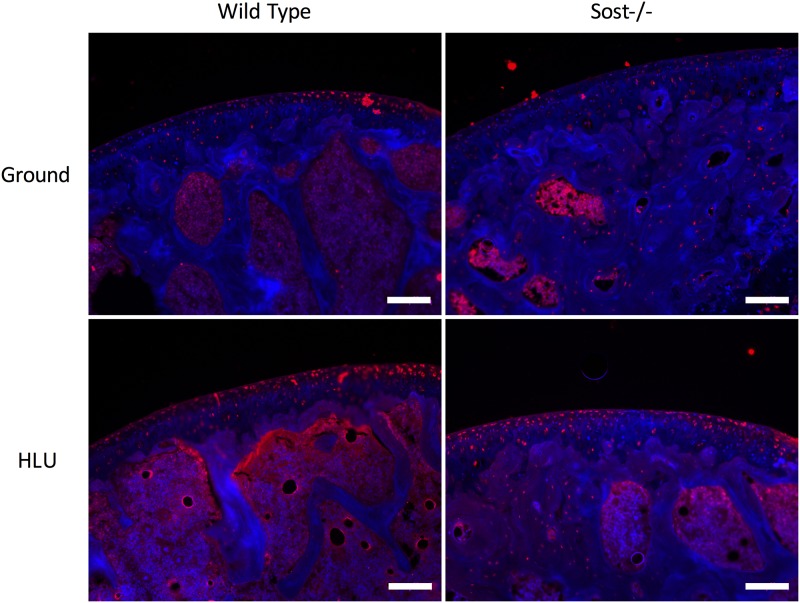
Immunofluorescence of LRP5 in response to hindlimb suspension. LRP5 expression in the articular cartilage of mouse femoral heads after 14 days of hindlimb suspension. All scale bars = 100 μm.

Because of the high LRP5 upregulation observed in both our RT-PCR and *in vitro* immunostaining assays, we tested whether LRP5 was also modulated *in vivo*. WT mice weighed 30.7±0.2 grams and Sost KO weighed 31.0±0.3 grams at the start of experiment. After two weeks of no hind limb suspension (HLS) control or HLS, WT control lost 1±0.2 grams body weight; WT HLS lost 4±0.2 grams; Sost KO lost 2.5±0.2 grams; and Sost KO HLS lost 6.7±1.0 grams. Microbiological status was not assessed. Mice did not receive drug treatment. There were no adverse events and there were no modifications to the experimental protocols made to reduce adverse events. For qualitative data, the staining was carried out in at least triplicate. We stained for LRP5 expression in the femoral heads of mice subjected to either normal loading or hindlimb suspension in both wild type mice and Sost-/- mice (Fig 4). In both wild type mice and Sost-/- mice LRP5 expression was elevated within the articular cartilage of mice subjected to hindlimb suspension relative to cartilage of the ground controls. No perceptible differences in LRP5 expression were observed between wild type and Sost-/- mice.

### Sclerostin modulates expression of LRP4 in a dose-dependent manner

**Fig 5:**
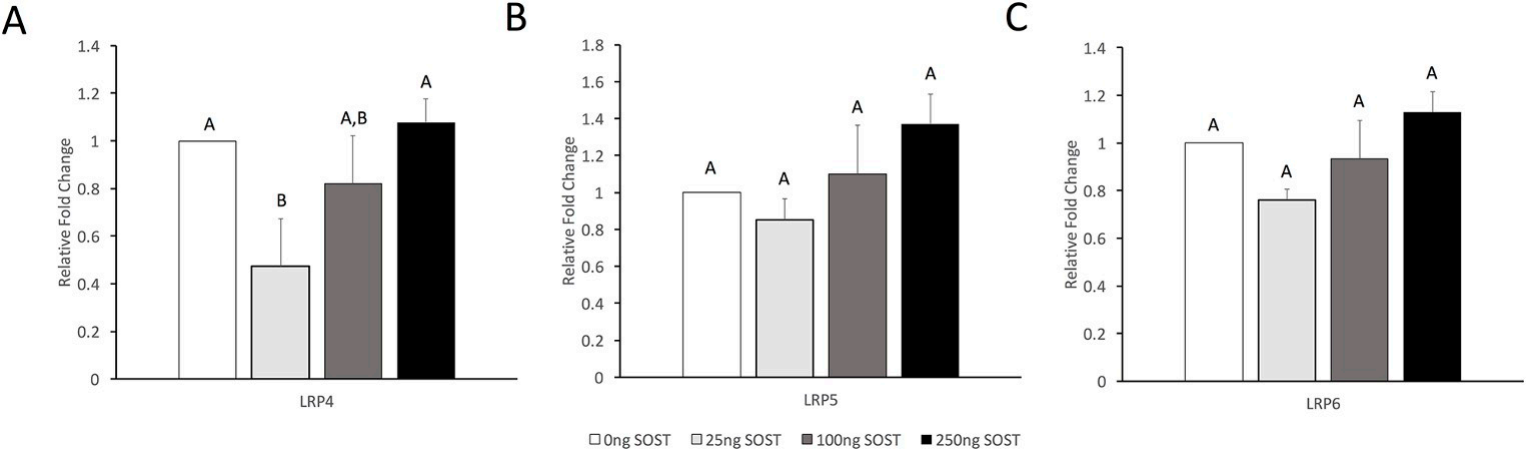
Gene expression of LRP4/5/6 in response to sclerostin. Expression of (**A**) LRP4, (**B**) LRP5, and (**C**) LRP6 after 48 hours of exogenous sclerostin. All data were normalized to the control 0 ng/ml sclerostin data represented by the dashed line. Levels not connected by the same letter are significantly different (p<0.05).

To determine the effect of sclerostin on LRP expression, RCS pellets were cultured in the presence of 25ng/ml, 100ng/ml, or 250ng/ml of recombinant sclerostin. Interestingly, a dose-dependent effect was observed (Fig 5). Of the three LRPs tested, LRP4 was most responsive to sclerostin addition and exhibited a reduction (0.47 fold) in expression with the addition of 25ng/ml sclerostin (p<0.05). Higher LRP4 levels were observed when sclerostin concentrations were increased to 100ng/ml and 250ng/ml, with statistical significance observed between 25ng/ml and 250ng/ml (p<0.05). LRP5 and LRP6 were not significantly affected.

## Discussion

The objective of this study was to investigate how simulated microgravity and cyclic hydrostatic pressure modulate chondrocyte homeostasis, specifically focusing on the effects of these mechanical stimuli on LRP4/5/6 expression. The Wnt-signaling pathway has been previously demonstrated to be an important regulator of musculoskeletal mechanobiology. We hypothesized that LRP4/5/6 expression is modulated via mechanical stimuli in the form of cyclic hydrostatic pressure and simulated microgravity. Our *in vitro* data demonstrated that LRP4/5/6 are regulated by the mechanical stimuli evaluated in this study. A deeper proteomic look at LRP5 confirmed that LRP5 is upregulated in both simulated microgravity and cyclic hydrostatic pressure. In addition, LRP5 immunohistological staining intensity was greater in the articular cartilage of hindlimb suspended mice relative to articular cartilage of the ground controls *in vivo*. This is the first study to demonstrate the response of LRPs to mechanical stimulation in chondrocytes.

Varying pellet sizes were observed in response to the mechanical stimuli. Pellets maintained in simulated microgravity grew larger than pellets maintained in control or cyclic hydrostatic pressure conditions. This is consistent with previous studies that observed larger cartilage pellets or aggregates cultured in RWV bioreactors when compared to static controls, likely due to the dynamic laminar flow in rotating bioreactors and increased nutrient diffusion [53–55]. However, we previously tested the gene expression of human adipose derived stem cells cultured in microgravity, static culture or cyclic hydrostatic pressure, and found that aggrecan, sox9 and collagen II mRNA expression was upregulated in cyclic hydrostatic pressure, suggesting that simulated microgravity decreased chondrogenesis when compared to pellets cultured under cyclic hydrostatic pressure conditions [22]. These historic findings suggest that cells may divide more rapidly in rotating bioreactors, the chemical and mechanical properties of the end-product reflect native cartilage tissue more when cultured under hydrostatic pressure conditions.

LRP5 is necessary for skeletal mechanotransduction [56]. The trabecular bone of LRP5 knockout mice has greater sensitivity to disuse [57]. Previous reports on LRP5 mRNA expression in response to mechanical loading have been contradictory. Lau *et al*. demonstrated an upregulation of LRP5 mRNA *in vitro* in osteoblasts subjected to fluid shear and an upregulation of LRP5 mRNA *in vivo* in the tibia of mice subjected to a four-point bending exercise regimen [58]. However, Robinson et al. reported no change in LRP5 or LRP6 mRNA expression *in vivo* when mice were loaded using the same method [2]. To our knowledge, this study and our findings are the first to suggest that LRP5 is involved in cartilage mechanobiology. The role of LRP5 within cartilage has been controversial. LRP5 deletion has been found to increase cartilage degeneration in osteoarthritic mouse models [59]. However, activated Wnt signaling has been reported to have a catabolic effect on cartilage tissue and inhibition of Wnt signaling through sclerostin has been found to be chondroprotective [60]. Hence, it is expected that excessive LRP5 expression would lead to cartilage degeneration. Within this study, LRP5 expression was elevated in response to both cyclic hydrostatic pressure and simulated microgravity conditions. LRP6, a protein that has been reported to have at least a partially redundant role to LRP5 [61], followed the same trend. *In vivo*, a similar mechano-response was observed in our hindlimb suspension model. Suspended mice displayed an increase in articular cartilage LRP5 expression, which suggests that unloading conditions enhance Wnt-signaling transduction in cartilage.

In this study we also observed changes in LRP4 of RCS pellets when exposed to either two weeks of cyclic hydrostatic pressure or simulated microgravity. LRP4 expression decreased at day 7 and day 10 relative to the 0-day control and 3-day time point. While LRP4’s role within the Canonical Wnt-signaling pathway has yet to be fully elucidated, studies have implicated that LRP4 may act as a sink for Wnt antagonists such as sclerostin and Wise [62,63]. The observed repression of LRP4 at day 7 might be a protective response initiated by the cell to release more sclerostin and counteract the large spike in LRP5 expression observed at day 3. Further investigation is needed to fully elucidate LRP4’s role within cartilage mechanobiology.

In this study, we did not observe nuclear translocation of β-catenin in response to mechanical stimulation, but this is consistent with previous literature. Normal human chondrocytes demonstrated cytoplasmic localization after 3 hrs of cyclic hydrostatic pressure, and the nuclear localization of β-catenin in OA chondrocytes was reduced after cyclic hydrostatic pressure [64]. In a study that examined the synergistic effect of tensile strain and Wnt3a signaling on nuclear translocation of β-catenin it was reported that tensile strain did not trigger complete nuclear translocation of β-catenin, but Wnt3a did stimulate nuclear translocation of β-catenin [40]. When chondrocytes were stimulated with both tensile strain and Wnt3a only partial nuclear translocation of β-catenin was observed immediately following loading period. Once the chondrocytes stimulated with both tensile strain and Wnt3a were allowed to recover for four hours, many of the cells did exhibit nuclear translocation of β-catenin [40]. In the ATDC5 cell line it was demonstrated that the number of cells positive for nuclear β-catenin was dependent on the duration of stretch loading [65]. Taken together, the literature suggests there is likely a complex, time-dependent relationship between nuclear translocation of β-catenin and mechanical stimulation that is not fully understood and should be studied in further depth in future studies.

The observed changes in LRP expression could be caused directly by mechanical stimulation, indirectly by stimulation through another mechanoresponsive protein, or a combination of both. Here we examined how sclerostin, an antagonist of Wnt signaling, regulates LRP4/5/6 expression in cartilage. Sclerostin appears to be a major regulator of bone’s response to mechanical loading [66]. It acts by binding to the LRP5/6 receptors, effectively blocking the Wnt signaling from transducing intracellularly [66]. It has been shown that serum levels of sclerostin and DKK1, another Wnt-inhibitor, are elevated in patients on bed-rest [67,68]. Mechanical loading has been shown to reduce sclerostin expression, while unloading increases sclerostin expression in bone [69]. and the down regulation of sclerostin has been shown to be necessary for increased bone formation in response to mechanical loading [70]. Sclerostin has also been demonstrated to be upregulated in simulated microgravity in osteocytes and this upregulation has been shown to be cell autonomous in osteocytes and hence independent of endocrine or paracrine factors [71]. This is consistent with studies that have shown that mechanical unloading of bone increases sclerostin expression [72]. Additionally, sclerostin inhibition has been reported to prevent loss of bone due to unloading [72,73]. While sclerostin is unfavorable to bone density maintenance, it appears to act as a defense mechanism to modulate Wnt signaling and prevent excessive cartilage degradation [60].

In this study, we found that the addition of exogenous sclerostin to chondrocytes modulated LRP4 expression, but the modulation of LRP5/6 expression was not statistically significant. While lower concentrations of sclerostin were found to repress LRP4 expression, higher concentrations of sclerostin restored LRP4 expression patterns to non-treated levels, likely due to saturation. Sclerostin has been previously reported to repress LRP5 and LRP6 expression in ovine chondrocytes using the same range of sclerostin concentrations [60]. The discrepancy could potentially be because chondrocytes were used from different animal models, which may have different physiologic concentrations of sclerostin. Additionally, it has been well documented that rats and other small animal models have a greater intrinsic capacity to cartilage regeneration than large animal models [74,75], which suggests that altered mechanism may be observed between ovine and murine models. While small animal models, such as the hindlimb suspension model, may be useful for identifying mechanisms of interest, these data must be verified in large animal studies before the development of human pharmaceuticals can arise. In the current study, we also compared LRP5 staining between Sost-/- mice and wild type mice and observed similar staining patterns, which supports the modest changes in LRP5 expression observed with the addition of exogenous sclerostin *in vitro*. Nevertheless, results from this study suggest that sclerostin modulates LRP4 expression and, in conjunction with previous research showing that mechanical loading regulates sclerostin expression [67–69,71], changes in sclerostin expression in response to mechanical stimuli may still partially contribute to observed LRP expression patterns. Hence, sclerostin should be further evaluated as a pharmaceutical target for osteoarthritis treatment in the case of abnormal loading patterns experienced by patients on long-term bed rest or astronauts on long-term space missions.

A limitation of the present study is that the in vivo studies only examine two conditions: ground control and hindlimb suspension. Future studies should examine how supra-physiological loading regulates LRP receptors in vivo through the use of methods such as in vivo tibial compression. Another aspect that would be of interest to future studies would be further investigating if the observed changes in expression of LRP receptors lead to downstream effects within the Wnt-signaling pathway. Since LRP4/5/6 are receptors in the Wnt-signaling pathway [63,76], it is expected that the elevated LRP expression patterns observed here would correspond to upregulation of Lef1, c-fos, MMPs, and other Wnt targets. Furthermore, it would be of great interest to study how LRP4/5/6 knockout models respond to mechanical stimuli in terms of matrix gene expression and matrix deposition. Although these studies were outside the scope of the present work, they should be explored in future studies.

This is the first study to show that LRP4/5/6 expression is modulated via mechanical stimulation. Specifically, we have demonstrated that RCS pellet growth was modulated by mechanical stimulation. LRP4/5/6 expression is affected in chondrocytes exposed to either cyclic hydrostatic pressure or simulated microgravity. Both simulated microgravity and cyclic hydrostatic pressure increase LRP5 expression. LRP5 expression is also increased in an *in vivo* mouse hindlimb suspension model. Finally, we have shown that exogenous sclerostin modulates the expression of LRP4. Further elucidation of the role that mechanical stimulation modulates the Wnt-signaling pathway could lead to development of effective countermeasures against cartilage degeneration due to overuse or disuse. However, it should be noted that while active Wnt-signaling may have adverse side effects in cartilage [60], it is known to be beneficial to bone development and remodeling [77]. This research warns of the potential side effects that current SOST-targeting osteoporosis treatments may have on the neighboring cartilage. Better understanding mechano-modulation of the Wnt-signaling pathway will be critical to determining if and how the Wnt-signaling pathway should be modulated pharmaceutically to best promote both bone and cartilage health.
